# Clinical Characteristics and Laboratory Identification of* Aerococcus* Infections: An Australian Tertiary Centre Perspective

**DOI:** 10.1155/2017/5684614

**Published:** 2017-09-13

**Authors:** Shanti Narayanasamy, Katherine King, Amanda Dennison, Denis W. Spelman, Ar Kar Aung

**Affiliations:** ^1^Department of Infectious Disease, The Alfred Hospital, Melbourne, VIC, Australia; ^2^Microbiology Unit, The Alfred Hospital, Melbourne, VIC, Australia; ^3^Department of General Medicine, The Alfred Hospital, Melbourne, VIC, Australia; ^4^Monash University, Melbourne, VIC, Australia

## Abstract

Aerococci uncommonly cause urinary tract (UTI) and bloodstream infections (BSI). The clinical characteristics and laboratory identification rates of* Aerococcus* in the Australian context are unknown. A retrospective observational cohort study of patients with positive* Aerococcus *cultures between 2010 and 2015 was performed. Patients were analysed according to predefined “asymptomatic bacteriuria,” “UTI,” and “BSI” groups. Forty-seven [40 (85%) for urine and 7 (15%) for blood] isolates were identified [38% male, median age of 79 (IQR 62–85) years], with corresponding identification rates of 24.2/100,000/year for urine (0.02%) and 7.3/100,000/year for blood cultures (0.007%). Since the use of matrix-assisted laser desorption ionisation time-of-flight mass spectrometry (MALDI-TOF MS) identification rate in urine has increased from 14.7/100,000/year to 32/100,000/year (*p* = 0.02). For urine isolates, 14 (35%) met the definition for UTI whilst 26 (65%) were “asymptomatic bacteriuria.” Underlying urological abnormalities, catheterisation, and polymicrobial growth were common. Seventy percent of bacteriuria was treated regardless of colonisation or active infection status. Symptomatic patients were more likely to receive treatment (OR 7.2, 95% CI 1.4–35.3). In patients with BSI, 1 (14.2%) had endocarditis and 1 (14.2%) died. The majority of isolates were susceptible to penicillin (11/12 tested, 92%).

## 1. Background

*Aerococcus *species were first identified in 1953 as catalase negative, alpha-haemolytic, Gram positive cocci distinct from streptococci and enterococci [1]. Since then, five species have been identified as pathogenic in humans, with a spectrum of disease ranging from urinary tract infections (UTI), bloodstream infections (BSI) [2], and endocarditis [3–5] to spinal infections [6, 7]. Laboratory identification of* Aerococcus *species has evolved over the last decade with the advent of matrix-assisted laser desorption ionisation time-of-flight mass spectrometry (MALDI-TOF MS) technology [8], providing improved sensitivity and accuracy in species identification, leading to further understanding of their role in clinically significant infections [9].

Recent studies have estimated the incidence of* Aerococcus *in urine to be between 0.15 and 0.27% of cultures sent to the laboratory [2, 10, 11].* Aerococcus *bacteriuria is known to be common amongst the elderly, with a female predominance [9]. The majority of urinary isolates are* Aerococcus urinae *(55–66%) followed by* Aerococcus sanguinicola *(26–46%).* Aerococcus viridans *is uncommon [9]. Aerococci are also presumed to be part of normal genitourinary tract flora, and isolation in urine may not necessarily represent clinical infection. Invasive diseases, including bloodstream infections, are, however, uncommon. The understanding of clinical significance of* Aerococcus* in sterile site cultures may be limited amongst clinicians. To date, clinical characteristics and identification of* Aerococcus* infections in Australian context have not been described.

The primary aims of this study were to describe the clinical characteristics of patients in whom* Aerococcus* species was isolated from a sterile site (urine and blood cultures) at an Australian tertiary hospital. Further, we sought to compare the differences between patients who met strict clinical and laboratory criteria for urinary tract infection (UTI) and those who were deemed to have urinary “asymptomatic bacteriuria” with* Aerococcus*. Secondary aims were to review the laboratory identification rates of* Aerococcus* species in clinical samples over time and to determine whether MALDI-TOF MS technology altered the identification rates compared with previous laboratory methods.

## 2. Methods

We conducted a retrospective, observational cohort study on all patients with* Aerococcus *isolates in blood or urine samples over a period of five years and five months from 1st of January 2010 to 31st of May 2015 at The Alfred Health Microbiology Laboratory, servicing three university affiliated hospitals, The Alfred Hospital, Caulfield Hospital, and Sandringham Hospital (total 800 beds) in metropolitan Melbourne, Australia. The Alfred Health affiliated network of hospitals serve a diverse group of patients, including community referrals, medical and surgical subspecialities, solid organ and bone marrow transplant, burns, HIV, psychiatry, and rehabilitation. This study was approved by Alfred Health Ethics Committee (approval number 301/15).

### 2.1. Clinical Data

Demographic and clinical data were obtained from hospital electronic records. These included age, sex, major comorbidities predisposing to infections (such as diabetes mellitus, known urological disease/abnormality, renal disease, cardiac disease, stroke, impaired cognition), episodes of UTI within one year before, immunocompromised status, urinary catheter status prior to cultures, any urological procedures 30 days before, central venous catheter (CVC) access within seven days before, presence of fever (temperature > 37.9°C) or hypothermia (<35.5°C), serum total white cell count and neutrophil differential, C-Reactive Protein (CRP) levels, presence of polymicrobial growth in cultures, and antimicrobial susceptibilities. Clinical outcome data included Intensive Care Unit (ICU) admission during the inpatient episode, antimicrobial treatment and route, mortality, complications, and relapse.

The age-adjusted Charlson Comorbidity Index [12] was also calculated for each patient. Healthcare associated infection/colonisation was defined as onset of infection/colonisation with positive cultures >48 hours after hospital admission. Immunocompromised status was defined as solid organ transplant recipient, haematological stem-cell transplant recipient, autoimmune/connective tissue disorder, known malignancy, or recipient of >10 mg prednisolone daily for >one month.

All those with positive blood culture isolates were considered to have a bloodstream infection (BSI), whereas patients with positive urinary isolates were characterised as either having a “UTI” or having “asymptomatic bacteriuria” based on predetermined clinical and microbiological criteria. Patients were considered to have a UTI if they met both of the following microbiological criteria: (1) presence of ≥50 × 10^6^ white blood cells/L on urine microscopy and (2) organism count of >100 × 10^6^/L on urine culture. They were also required to have at least one of the following clinical symptoms: urinary tract symptoms (dysuria, frequency, new nocturia, new incontinence, new urinary retention, suprapubic pain, and flank pain), fever/hypothermia, or systemic symptoms (myalgias, chills, altered mental status/delirium, and hypotension) [11, 13]. Patients with* Aerococcus *urine isolates who did not meet the microbiological and clinical criteria were considered “asymptomatic bacteriuria.”

### 2.2. Microbiological Data

A quantitative culture of urine was performed with a 1 *μ*L loop of specimen on Oxoid Columbia agar with 5% horse blood and Oxoid MacConkey agar Number 3. Specimens were incubated overnight at 35°C in air.

Blood was cultured using the BacT/ALERT® 3D system (bioMérieux, Marcy l'Etoile, France). Five to ten millilitres of blood was inoculated into aerobic and anaerobic culture bottles. Upon receipt in the laboratory, the bottles were incubated at 35°C for a total of five days or until they were flagged as positive by the instrument. Upon flagging, a Gram stain was performed. Bottles showing Gram positive cocci were inoculated onto Oxoid Columbia agar with 5% horse blood and incubated at 35°C in 5% CO_2_ until sufficient growth for organism identification was observed.

Standard referencing guidelines were used for urine interpretations, where* Aerococcus* species are considered an “unusual but significant urinary pathogen” [14]. If* Aerococcus* was the only organism isolated and the organism count was 10–100 × 10^6^/L, identification to the species level was performed (where possible). In polymicrobial urine samples,* Aerococcus* species needed to be present in numbers of >100 × 10^6^/L and outnumber all other microbiota by at least 10-fold for identification to be performed.

Prior to the introduction of MALDI-TOF MS (VITEK-MS, bioMérieux, Marcy l'Etoile, France) in August 2012, preliminary identification of* Aerococcus* to the genus level was based on colony morphology, Gram stain morphology, and catalase reaction. Alpha-haemolytic colonies showing Gram positive cocci in clusters with a weakly positive catalase reaction were further identified using either the bioMérieux API-20 Strep or bioMérieux VITEK GP card. These systems are only able to identify either* Aerococcus urinae* or* Aerococcus viridans* to the species level. Non-*viridans* or* urinae* Aerococci were simply reported as* Aerococcus *species.

After the introduction of MALDI-TOF MS, Aerococci continued to be preliminarily identified based on colony morphology and Gram stain morphology. Isolates found to be alpha-haemolytic Gram positive cocci were formally identified using the MALDI-TOF. Unlike the Bruker MALDI Biotyper, the VITEK MALDI-TOF MS can only identify* Aerococcus viridans* and* Aerococcus urinae* to a species level. All other Aerococci are only able to be identified to genus level. Blood isolates that were unable to be identified beyond the genus level were referred to the Microbiological Diagnostic Unit at Melbourne University for further species identification using 16sRNA molecular testing.

Antibiotic susceptibilities were performed with *E* test strips (bioMérieux) to generate a Minimum Inhibitory Concentration (MIC). Results were reported as the MIC value only. No interpretations were provided as breakpoints were not available through either the European Committee on Antimicrobial Susceptibility Testing (EUCAST) or the Clinical Laboratory Standards Institute (CLSI) guidelines at the time of susceptibility testing. Interpretation of MIC results was at the discretion of the clinician.

### 2.3. Statistical Analysis

All categorical data were presented as counts and proportions whilst continuous data were presented as median and interquartile ranges. Incidence rate was calculated as the number of positive isolates per 100,000 urine or blood culture samples received in a year. Chi-square, Fisher's exact tests, and Mann–Whitney *U* tests were employed for comparisons in incidence rates before and after MALDI-TOF and between “UTI” and “asymptomatic bacteriuria” groups, using GraphPad Prism 6. A two-sided *p* value of <0.05 is considered statistically significant for all associations.

## 3. Results

### 3.1. Clinical

A total of 47* Aerococcus* isolates [40 (85%) for urine and seven (15%) for blood] were identified over the study period. Of these,* A. viridans* was isolated in 28 (60%),* A. urinae *in 17 (36%), and* Aerococcus *species (not speciated) in 2 (4%).

Table 1 provides demographic and clinical data for patients with positive urine and blood isolates. A majority of those with bacteriuria (both UTI and “asymptomatic bacteriuria”) were elderly (≥65 years) females whereas those with positive blood isolates were younger males. Healthcare associated infections were common in all three conditions. Underlying urological conditions and urinary catheterisation were frequently noted in patients with bacteriuria (in >25% of patients).

Polymicrobial growth was common amongst the patients with bacteriuria [a total of 14 (35%) had polymicrobial growth, four in the “UTI” group, and 10 in the “asymptomatic bacteriuria” group] (Table 2). Of those, seven (50%) had concurrent growth with* Escherichia coli*, five (36%) had other Gram negative enteric flora, and two (14%) had* Enterococcus faecalis*.

Of patients with BSI, only one (14%) was immunocompromised. None of those with BSI had a central venous access prior to onset of bacteraemia. Notably, none of those with BSI isolated the same organism in urine and none had clinical features consistent with concurrent UTI. Of those with positive blood cultures, three (43%) had polymicrobial growth, two with coagulase negative staphylococci and one with a* Streptococcus *species.

When strict predefined criteria were applied to urine isolates, only 14 (35%) met the definition for UTI, whilst 26 (65%) met criteria for “asymptomatic bacteriuria.” The majority (70% overall) of those with positive urine cultures received antimicrobial therapy, 11 (79%) in those who met predefined criteria for UTI and 17 (68%) in those with “asymptomatic bacteriuria” (Table 2). The “asymptomatic bacteriuria” patients were more likely to be treated with oral antibiotics alone (71% versus 18%, *p* = 0.018). However, those who fulfilled strict clinical and laboratory criteria for UTI were more likely to be treated with intravenous nonpenicillin antibiotics compared to the “asymptomatic bacteriuria” patients (Table 2). Overall, symptomatic patients were more likely to receive treatment (OR 7.2, 95% CI 1.4–35.3, and *p* = 0.015).

Amongst those with blood isolates, one (14%) had endocarditis and one (14%) died of sepsis (not due to* Aerococcus* infection). There were no cases of relapsed or recurrent* Aerococcus* infection.

### 3.2. Laboratory Findings

**Figure 1:**
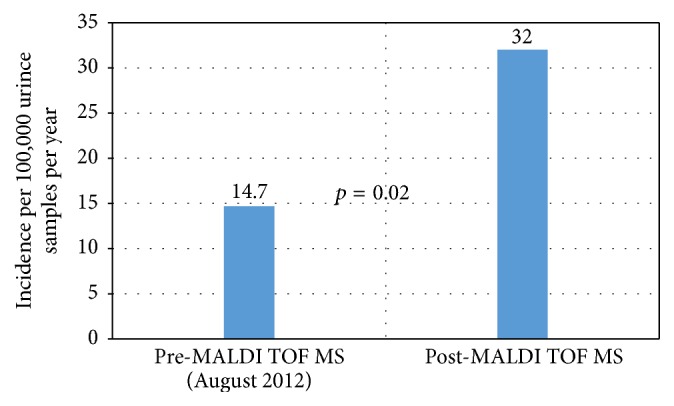
Rate of* Aerococcus* species isolation in urine before and after introduction of MALDI-TOF. (Pre-MALDI-TOF MS: 1st of Jan 2010–August 2012; Post-MALDI-TOF MS: August 2012 to 31st of May 2015).

Over the study period, the Alfred Microbiology Laboratory received 165,511 urine and 96,332 blood specimens for culture during this time. This corresponds to an identification rate of 24.2 per 100,000 samples per year (0.02%) for urine and 7.3 per 100,000 samples per year (0.007%) for blood cultures, respectively. Since the use of MALDI-TOF in our laboratory in 2012, the identification rate for* Aerococcus* in urine has increased from 14.7 per 100,000 per year to 32 per 100,000 per year (*p* = 0.02) (Figure 1).

Twelve (26%) isolates were tested for susceptibilities with 11 (92%) susceptible to penicillin or ampicillin at median MIC of 0.032 mg/L. One isolate, an* A. viridans* in urine was resistant to penicillin (MIC > 32 mg/L) and ceftriaxone (>32 mg/L).

## 4. Discussion

To date,* Aerococcus* remains an organism whose epidemiology and clinical characteristics are not yet fully understood. This retrospective cohort study explored the clinical characteristics of* Aerococcus* infections in an Australian context, at a major tertiary institution serving a diverse group of patients ranging from community dwellers to hospitalised immunocompromised patients. Moreover, it highlighted the critical role of MALDI-TOF MS technology in improving laboratory identification rate of* Aerococcus*, which may further aid in evolving understanding on clinical and epidemiological aspects of this uncommon organism.

In keeping with previous studies, this study demonstrated that both symptomatic and asymptomatic bacteriuria were common in older women, whereas bacteraemia occurred in males of slightly younger age group [2, 9, 11, 15]. It also found that a moderate proportion of patients with bacteriuria had known urological conditions (35%) or prior urinary catheter insertion (>25%), similar to previously published data [11]. Additionally, a high proportion of patients were also noted to have preexisting diabetes, cognitive impairment, and healthcare acquisition as possible risk factors for bacteriuria.

We found that up to two-thirds of patients with bacteriuria did not meet the strict predefined clinical criteria for UTI, suggesting that* Aerococcus* is a common coloniser of urine. Despite this, a majority of patients (~70% overall) were treated with antimicrobials based on culture results alone, differing from local and international guidelines, which do not recommend antimicrobial treatment [13, 16]. Differential approaches to treatment were also apparent, with those in the “asymptomatic bacteriuria” group more likely to receive oral penicillins, whilst those with symptomatic infection were more likely to receive intravenous nonpenicillin antibiotics, such as ceftriaxone. This perhaps reflects the lack of familiarity with* Aerococcus* and, therefore, clinicians' choice to provide dedicated Gram negative cover for symptomatic urine infection.

*Aerococcus* bacteraemia is known to be associated with concurrent UTI as a possible primary source [15], with male gender, older age, underlying urological conditions, and catheterisation being risk factors [5]. In contrast, bacteraemic patients in this study had no clinical or microbiological evidence of concurrent UTI, which, notwithstanding the small number of isolates, raises the possibility of an unrecognised nonurinary source (i.e., gastrointestinal focus).

In our laboratory, the overall identification rates of* Aerococcus* in urine samples was 0.02%, a figure tenfold lower than those published in the literature [9, 11]. One explanation for this is the difference in thresholds for reporting based on CFU between our laboratory and European or American laboratories, where the previously published incidence data originated. We inoculate 1 *μ*L of urine on the culture medium and consider growth of >10–100 × 10^6^/L (i.e., >10^7^–10^8^ CFU/L) as significant, in accordance with the American Society for Microbiology Cumulative Techniques and Procedures in Clinical Microbiology Handbook [17]. However, previous studies on* Aerococcus* employed a higher inoculum at 10 *μ*L of urine on culture medium and a lower report threshold of >10^7^ CFU/L for significant growth, which is at least 10-fold lower than our methods [2, 18].

We found that most of the local isolates were of* A. viridans* or* A. urinae*. This is in contrast to previous studies from other regions (Europe and the United States) which demonstrated that* A. sanguinicola* was prevalent whilst* A. viridans* was uncommon [2, 9]. Prior to the use of MALDI-TOF MS,* Aerococcus* identification in our laboratory utilised colony morphology, Gram stain results, and catalase reactions. By this method, many isolates were probably mistakenly identified as alpha-haemolytic* Streptococcus* or* Granulicatella* species, especially in the presence of other contaminating flora [19]. Additionally, for* Aerococcus* isolates which achieved genus level identification, further accurate species level identification proved to be error-prone due to limitations by API-20 Strep or VITEK GP card methods, with the possibility that* A. sanguinicola* isolates were assigned as* A. viridans *[20]. Collectively, this may account for low identification rates of* Aerococcus* during pre-MALDI-TOF period and also higher than expected rates for* A. viridans* compared to other published data.

Introduction of MALDI-TOF MS in our laboratory increased the rate of* Aerococcus* identification by at least tenfold, in keeping with previously published data employing MALDI-TOF MS method [2]. The Bruker MALDI Biotyper has been demonstrated to reliably identify a broad range of* Aerococcus* species:* A. christensenii*,* A. sanguinicola, A. urinae, A. urinaehominis, *and* A. viridans* [20]. However, species identification continued to be problematic after introduction of MALDI-TOF MS as the VITEK system employed in this study can only identify two species* A. viridans* and* A. urinae *reliably [21]. Therefore, whether our results represent ongoing challenges in laboratory identification of* Aerococcus* species or a true geographical variation in species distribution needs to be further elucidated.

Our study reflects the literature that the majority of* Aerococcus *species display low MICs to penicillin or ampicillin, which is the treatment of choice [2, 22]. Recently, both EUCAST and CLSI have introduced susceptibility breakpoints for* Aerococcus *spp. by both MIC and disk diffusion methods (EUCAST version 7.1 and CLSI M-45 A3) [23, 24]. Employing the EUCAST MIC breakpoints of ≤0.125 mg/L for benzylpenicillin and ≤0.25 mg/L for ampicillin, we found that >90% of isolates tested were susceptible.

This study was limited by its small sample size and observational and retrospective nature. Despite being single laboratory-based study, the diversity of patient populations included was considerable, ranging from community to specialist care episodes. Notwithstanding aforementioned issues with species identification, our study provided some useful insights into the local prevalence, clinical characteristics, and further highlighted major challenges in the laboratory diagnosis of* Aerococcus*. To date, there is limited evidence as to whether different* Aerococcus* species influence differential clinical presentations. As such it is probably valid to analyse the clinical characteristics of* Aerococcus* species as a whole, our main intent. Nevertheless, molecular typing methods such as 16S-rRNA sequencing or the utilisation of the Bruker MALDI Biotyper system for exact species identification are likely to be helpful in determining the epidemiology and clinical correlations in future studies. Although this study provides a limited snapshot of* Aerococcus* in the Australian context, robust epidemiological data supported by standardised molecular species identification methods through collaborative efforts involving major laboratories across the continent may yet provide a better data on the local epidemiology of this uncommon organism.

## 5. Conclusions

*Aerococcus* remains as an uncommon and unfamiliar organism to many clinicians with varied treatment approaches, particularly for bacteriuria, despite high rates of penicillin susceptibility. Therefore, recognition of important clinical features and risk factors is key in consideration of treatment. Overall, MALDI-TOF MS has been shown to improve the identification rates of* Aerococcus* from sterile site samples. However, given the low frequency of this organism, large collaborative studies between laboratories employing standardised isolation and identification methods, including routine use of MALDI-TOF MS technology and molecular identification strategies, are needed to estimate the true regional prevalence and disease burden in Australia.

## Figures and Tables

**Table 1:** Demographics: UTI, “asymptomatic bacteriuria,” and bloodstream infection.

	UTI (*N* = 14)	Colonized (*N* = 26)	BSI (*N* = 7)
Age in years (median, IQR)	81.5 (54.8–87.3)	80 (69–87)	62 (52–78)
Age ≥ 65 years (*n*, %)	9 (64.3)	21 (80.8)	3 (42.9)
Male (*n*, %)	4 (28.6)	9 (34.6)	5 (71.4)
Inpatient (*n*, %)	8 (57.1)	17 (65.4)	6 (85.7)
Healthcare acquired (*n*, %)	10 (71.4)	15 (57.7)	5 (71.4)
Immunocompromised (*n*, %)	1 (7.1)	6 (23.1)	1 (14.3)
*Co-Morbidities*			
Diabetes (*n*, %)	3 (21.4)	8 (30.8)	2 (28.6)
Urological conditions (*n*, %)	4 (28.6)	10 (38.5)	3 (42.9)
Renal disease (*n*, %)	3 (21.4)	2 (7.7)	2 (28.6)
Cardiac disease (*n*, %)	5 (35.7)	9 (34.6)	4 (57.1)
Stroke (*n*, %)	1 (7.1)	6 (23.1)	1 (14.3)
Impaired cognition (*n*, %)	5 (35.7)	11 (42.3)	0 (0.0)
Age-adjusted Charlson Co-morbidity Index (median, IQR)	5 (2.8–6.1)	5 (4–6.3)	5 (2–7)
UTI within last one year (*n*, %)	4 (28.6)	4 (15.4)	0 (0.0)
*Indwelling Catheter Status*			
Permanent (*n*, %)	2 (14.3)	3 (11.5)	0 (0.0)
Transient (*n*, %)	2 (14.3)	4 (15.4)	4 (57.1)
Urological procedures 30 d prior (*n*, %)	1 (7.1)	1 (3.8)	0 (0.0)

BSI: bloodstream infections, CVC: central venous catheter, IDC: indwelling urinary catheter, IQR: interquartile range, UTI: urinary tract infection.

**Table 2:** Outcomes: UTI versus “asymptomatic bacteriuria.”

	UTI (*N* = 14)	Colonized (*N* = 26)	*p* value
*Clinical and Laboratory Parameters*			
Presence of fever, ≥37.9°C or hypothermia, <35.5°C (*n*, %)	2 (15.4)^a^	6 (27.3)^c^	0.68
White cell count, ×10^9^ per litre (median, IQR)	8.9 (6.0–16.9)^a^	9 (6.3–10.6)^a^	0.67
Neutrophil count, ×10^9^ per litre (median, IQR)	6.1 (4.2–13.9)^a^	6.2 (3.7–8.9)^a^	0.37
CRP, mg/L (median, IQR)	16.5 (8.0–88.8)^b^	17 (7–63)^d^	0.97
Polymicrobial growth in urine (*n*, %)	4 (28.6)	10 (38.5)	0.73
*Treatment* ^e^			
Antimicrobial treatment (*n*, %)	11 (78.6)	17 (68.0)^a^	0.71
Intravenous only (*n*, %)	2 (18.2)	1 (5.9)	0.54
Oral only (*n*, %)	2 (18.2)	12 (70.6)	**0.018**
Both (*n*, %)	7 (63.6)	4 (23.5)	0.052
Penicillin use (*n*, %)	4 (36.4)	11 (64.7)	0.25
Other (*n*, %)	11 (100.0)	9 (52.9)	**0.010**
Duration of treatment, days (median, IQR)	9 (0.8–12)	4 (0–11.5)	0.21
*Outcomes*			
ICU admission (*n*, %)	0 (0.0)	2 (7.7)	0.53
Mortality, 3 months (*n*, %)	2 (20.0)^c^	5 (20.8)^b^	1.0
Complications (*n*, %)	0 (0.0)	1 (3.8)	1.0

CRP: C-reactive protein, ICU: intensive care unit, IQR: interquartile range, UTI: urinary tract infection; Data was not available for ^a^1, ^b^2  ^c^4 and ^d^11 subjects; ^e^Treatment was initiated in 11 subjects with UTI and 17 subjects with colonisation.

## Abbreviations

UTI: Urinary tract infections
BSI: Bloodstream infections
MALDI-TOF MS: Matrix-assisted laser desorption ionisation time-of-flight mass spectrometry
CVC: Central venous catheter
CRP: C-Reactive Protein
ICU: Intensive Care Unit.

## Data Access

The datasets used and/or analysed during the current study are available from the corresponding author on reasonable request.
